# Mitochondria-targeting polydopamine-coated nanodrugs for effective photothermal- and chemo-synergistic therapies against lung cancer

**DOI:** 10.1093/rb/rbac051

**Published:** 2022-08-01

**Authors:** Ziyu Meng, Binchao Wang, Yiqiang Liu, Yejian Wan, Qianshi Liu, Huasheng Xu, Renchuan Liang, Ying Shi, Peng Tu, Hong Wu, Chuan Xu

**Affiliations:** College of Science, Gansu Agricultural University, Lanzhou 730070, China; Integrative Cancer Center & Cancer Clinical Research Center, Sichuan Cancer Hospital & Institute, Sichuan Cancer Center, School of Medicine, University of Electronic Science and Technology of China, Chengdu 610047, China; College of Science, Gansu Agricultural University, Lanzhou 730070, China; Integrative Cancer Center & Cancer Clinical Research Center, Sichuan Cancer Hospital & Institute, Sichuan Cancer Center, School of Medicine, University of Electronic Science and Technology of China, Chengdu 610047, China; Integrative Cancer Center & Cancer Clinical Research Center, Sichuan Cancer Hospital & Institute, Sichuan Cancer Center, School of Medicine, University of Electronic Science and Technology of China, Chengdu 610047, China; Integrative Cancer Center & Cancer Clinical Research Center, Sichuan Cancer Hospital & Institute, Sichuan Cancer Center, School of Medicine, University of Electronic Science and Technology of China, Chengdu 610047, China; Integrative Cancer Center & Cancer Clinical Research Center, Sichuan Cancer Hospital & Institute, Sichuan Cancer Center, School of Medicine, University of Electronic Science and Technology of China, Chengdu 610047, China; Integrative Cancer Center & Cancer Clinical Research Center, Sichuan Cancer Hospital & Institute, Sichuan Cancer Center, School of Medicine, University of Electronic Science and Technology of China, Chengdu 610047, China; Integrative Cancer Center & Cancer Clinical Research Center, Sichuan Cancer Hospital & Institute, Sichuan Cancer Center, School of Medicine, University of Electronic Science and Technology of China, Chengdu 610047, China; College of Science, Gansu Agricultural University, Lanzhou 730070, China; Integrative Cancer Center & Cancer Clinical Research Center, Sichuan Cancer Hospital & Institute, Sichuan Cancer Center, School of Medicine, University of Electronic Science and Technology of China, Chengdu 610047, China; Integrative Cancer Center & Cancer Clinical Research Center, Sichuan Cancer Hospital & Institute, Sichuan Cancer Center, School of Medicine, University of Electronic Science and Technology of China, Chengdu 610047, China

**Keywords:** lung cancer, mitochondria-targeting, alpha-tocopherol succinate (α-TOS), chemo- and photothermal- synergistic therapies

## Abstract

Targeting mitochondria via nano platform emerged as an attractive anti-tumor pathway due to the central regulation role in cellar apoptosis and drug resistance. Here, a mitochondria-targeting nanoparticle (TOS-PDA-PEG-TPP) was designed to precisely deliver polydopamine (PDA) as the photothermal agent and alpha-tocopherol succinate (α-TOS) as the chemotherapeutic drug to the mitochondria of the tumor cells, which inhibits the tumor growth through chemo- and photothermal- synergistic therapies. TOS-PDA-PEG-TPP was constructed by coating PDA on the surface of TOS NPs self-assembled by α-TOS, followed by grafting PEG and triphenylphosphonium (TPP) on their surface to prolong the blood circulation time and target delivery of TOS and PDA to the mitochondria of tumor cells. *In vitro* studies showed that TOS-PDA-PEG-TPP could be efficiently internalized by tumor cells and accumulated at mitochondria, resulting in cellular apoptosis and synergistic inhibition of tumor cell proliferation. *In vivo* studies demonstrated that TOS-PDA-PEG-TPP could be efficiently localized at tumor sites and significantly restrain the tumor growth under NIR irradiation without apparent toxicity or deleterious effects. Conclusively, the combination strategy adopted for functional nanodrugs construction aimed at target-delivering therapeutic agents with different action mechanisms to the same intracellular organelles can be extended to other nanodrugs-dependent therapeutic systems.

## Graphical Abstract

**Figure:**
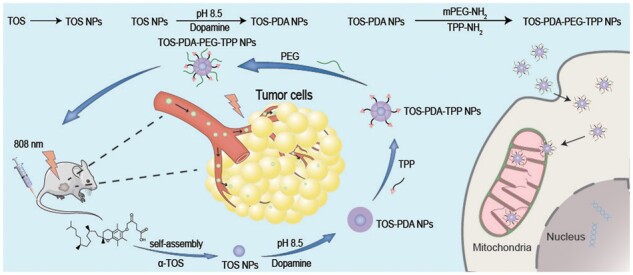

## Introduction

Lung cancer is a prevalent and lethal cancer worldwide. Although advances in therapeutic strategies have attracted much attention and various drugs have been developed in the past decades to prolong the survival of patients, the 5 years overall survival for advanced lung cancer patients is still < 15% [1–6]. The mitochondria of cancer cells are structurally and functionally different from their healthy counterparts, containing higher mitochondrial membrane potential, decreased oxidative phosphorylation, increased ROS production, increased mtDNA mutations, Ca^2+^ overload and failure to induce apoptosis [7–9]. Various studies have demonstrated that altered mitochondria functionality is a notable state of lung cancer and creates an exploitable vulnerability for cancer therapy [10, 11]. Hence, delivering anti-cancer drugs into mitochondria has become an attractive strategy for lung cancer therapy.

The anti-tumor effects of alpha-tocopherol succinate (α-TOS, a derivative of natural vitamin E) have been gradually discovered by targeting mitochondria to block the cell cycle, inhibit DNA synthesis and promote apoptosis or differentiation of tumor cells [12, 13]. Moreover, many studies have demonstrated that the integrated structure of α-TOS was crucial for its biological function. An urgent need to improve its anti-tumor effects was how to bring α-TOS to the mitochondrial of tumor cells in a complete form via the nano-carriers. Although mitochondria-targeting drug delivery systems have been developed in the past years, some drawbacks remain to be improved. The remarkable disadvantage is low drug-loading by carriers that could decrease treatment efficiency. To overcome the defect, we prepared carrier-free nanoparticles by α-TOS self-assemble and then modified them with functionalizing substances, making the nanoparticles with a higher drug-loading efficiency.

Nanocarriers decorated on the surface with triphenylphosphonium (TPP) are commonly used platform design in targeting the mitochondria of cells [14]. Except these, dopamine is a chemical that can self-polymerize on a variety of organic and inorganic materials by spontaneous oxidation in alkaline solutions (pH = 8.5) to form surface-adhesive polydopamine (PDA) films [15–18]. PDA shells are stable enough to reach target cells after intravenous injection. It is widely used as material surface modification [19–22]. Moreover, PDA membranes have extremely high advantages for photothermal therapy (PTT), which is a promising cancer treatment due to its low invasive burden and high spatiotemporal selectivity. In addition, PTT could rely on photothermal agents to convert the absorbed light energy into heat energy, resulting in the rise of local temperature and ultimately inducing the apoptosis of cancer cells [23–28].

In this work, we produced material for NIR-responsive PDA coating and an *in-situ* on-demand drug release system. α-TOS was used in self-assembled nanomedicines called TOS. A thin PDA film was then synthesized and exposed to the surface of the co-assembled TOS and TPP. We assumed that the TOS cycle time could be extended and the pre-leakage avoided by protecting PDA membranes. TOS passive tumor accumulation was promoted by the nanoparticles’ good enhanced permeability and retention (EPR) effect [29]. Upon transport to the tumor lesions, NIR radiation might destroy PDA films, and the loaded TOS NPs were released rapidly due to the strong NIR absorption and high photothermal conversion efficiency of PDA films [30–32]. Due to their inherent hydrophobicity, TOS NPs aggregated and slowed re-entry into the bloodstream, resulting in long-term local entrapment of the drug-carrier system within the tumor microenvironment [33–37]. The photothermal effect of PDA could trigger the release of small TOS NPs, which could penetrate deeply and evenly into cancer cells [38–42]. The mitochondrial targeting function of TPP could engulf the mitochondria of tumor cells, resulting in abnormal mitochondrial membrane potential of tumor cells and further promoting the rapid apoptosis of the cancer cells [43, 44]. The enrichment of TOS NPs to tumor sites, mitochondrial targeting and time control drug-releasing realized the systematic chemical and photothermal treatment of lung cancer.

## Materials and methods

### Materials

D-α-TOS was purchased from Sigma-Aldrich Co., Ltd (St. Louis, MO, USA). Dopamine hydrochloride (DA·HCl) was obtained from Shanghai Aladdin Biochemical Technology Co., Ltd (Shanghai, China). (2-Aminoethyl) triphenylphosphonium bromide (TPP-NH_2_) was bought from Accela ChemBio Co., Ltd (Shanghai, China). Tris(hydroxymethyl) aminomethane - and DMSO were purchased from Chengdu Kelong Chemical Co., Ltd (Chengdu, China). mPEG-NH_2_ was purchased from Shanghai ToYong Bio-Tech. Inc., China.

### Preparation of PDA-coated carrier-free nanodrugs

First, 50 mg α-TOS was dissolved into 2 mL DMSO solution followed by ultrasonic treatment for 10 min. Then, α-TOS solution was added dropwise into 5 mL of ultrapure water under stirring for 5 min and self-assembled into nanoparticles. Furthermore, the solution was dialyzed to remove DMSO against deionized (DI) water and then carrier-free nanoparticles (TOS NPs) solution was prepared. After that, 3 mL TOS NPs solution was mixed with 3 mL Tris-HCl solution (20 mM, pH 8.0) and then 1.2 mg DA·HCl was added to the mixture. After stirring at room temperature for 24 h, products were collected by centrifugal ultrafiltration (MWCO_3_KD) and washed with Tris-HCl solution (10 mM, pH 8.0) to remove the excess DA.

### Preparation of mitochondria-targeting nanoparticles

TPP (5 mg/mL, 100 µL) and mPEG-NH_2_ (2 mg) were added to TOS-PDA NPs (5 mL) solution for the synthesis of mitochondria-targeting nanoparticles. After stirring at room temperature for 24 h, the reaction solution was dialyzed (MWCO = 3500) against DI water to remove free reactants. Finally, we obtained the mitochondria-targeting nanoparticles (TOS-PDA-PEG-TPP NPs) solution. Similarly, the TOS-PDA-PEG NPs were prepared by adding mPEG-NH_2_ (2 mg) to the TOS-PDA NPs solution (5 mL). After stirring at room temperature for 24 h, the reaction solution was dialyzed (MWCO = 3500) against DI water to obtain the TOS-PDA-PEG NPs solution.

### Preparation of FITC-labeled nanoparticles

α-TOS and FITC were dissolved in DMSO and mixed together (ratio 10:1). The mixture was added dropwise into 5 mL of ultrapure water under stirring until the formation of nanoparticles. The solution was dialyzed to remove DMSO for 24 h against DI water and then FITC-labeled nanoparticles solution was prepared.

### Characterization of nanoparticles

The hydrodynamic diameter and zeta potential of nanoparticles were measured by a Malvern Zetasizer Nano ZS90 (Worcestershire, UK). To verify the modification of PEG and TPP, FT-IR spectrums of freeze-dried nanoparticles (TOS, TOS-PDA, TOS-PDA-PEG, TOS-PDA-PEG-TPP) were recorded between 4000 and 400 cm^−1^ on an FTIR spectrometer (PerkinElmer, USA). The morphologies of the nanoparticles were observed by transmission electron microscopy (TEM).

### Stability of nanoparticles

To assess the stability of nanoparticles, 500 µL nanoparticles solution was mixed with 500 µL fetal bovine serum (FBS). After 2, 6, 12, 24 and 48 h of incubation at room temperature, the mixture was photographed and measured size/zeta potential.

### *In vitro* photothermal effects

To study the photothermal effects of the nanoparticles, 1 mL nanoparticles solution (TOS, TOS-PDA-PEG, TOS-PDA-PEG-TPP) or 1 ml water was irradiated by the 808 nm laser with the power density of 1 W/cm^2^ for 5 min. To assess the photothermal constancy, the temperature curve of nanoparticles solution was measured after four-cycle on/off of NIR light. The temperature change of the solution was monitored by a thermal infrared camera and quantified by the FLIR Tools.

### Cell lines and animals

Human NSCLC cell line A549 and mouse lung Lewis carcinoma (LLC) cells were purchased from the ATCC and cultured in Dulbecco’s modified Eagle’s medium (CA, USA) supplemented with 10% FBS (Jiangsu, China) and 100 U/ml penicillin–streptomycin. The cells were cultured at 37°C incubator with 5% CO_2_ atmosphere. Male Balb/c mice (6–8 weeks) were purchased from Beijing Huafukang Bioscience Co. Inc and maintained in the Sichuan Cancer Institute. All animal experiments were performed in compliance with the Animal Ethics Committee of Sichuan Cancer Hospital.

### Nanoparticles uptake by cancer cells

Cancer cells were seeded into six-well plates with 5 × 10^5^ cells per well. After the cells adhered, nanoparticles were added, and the uptake of nanoparticles by cells was detected at 2 and 4 h, respectively. Confocal and flow cytometry were used to count the uptake of nanoparticles by cancer cells.

### Mitochondrial targeting and co-localization of the mitochondria

The LLC cells were inoculated in six-well plates with 5 × 10^3^ cells per well and were treated with different concentrations of nanoparticles. Mito-Tracker Red CMXROS was used according to the manufacturer’s instructions (Beyotime, C1049B, China).

### Cell cloning experiment

Cancer cells were seeded into six-well plates at 500 cells per well with a 2 mL complete medium. After 1 week of culture, drugs were added on the 8 th, 10 th and 12 th days, respectively. On the 14th day, the cells were fixed with 4% paraformaldehyde and stained with crystal violet for 15 min to take photos and count the clones.

### Cell proliferation assay

Cancer cells were cultured in 96-well plates at 4 × 10^3^ cells per well with a 0.2 mL complete medium. Different nanoparticles were added to the complete medium and cultured with cancer cells in a 37°C for 5 days. Cell Counting Kit-8 solution (Biosharp, BS350B, Anhui, China) was diluted with the medium at a ratio of 1:10 and incubated for 2 h. Optical density was measured based on absorbance at a wavelength of 450 nm.

### Mitochondrial membrane potential (ΔΨm) assay

LLC cells were treated with different nanoparticles for 24 h. The cells were washed twice with PBS, followed by JC-1 (Beyotime, C2003S, China) incubating at 37°C for 30 min in the dark. The fluorescence intensities were analyzed using confocal with the setting of 520 nm and 590 nm.

### Apoptosis assay

Apoptosis of cancer cells was detected with an Annexin V-FITC apoptosis detection kit (Beyotime, C1063, Shanghai, China) by flow cytometry according to the manufacturer’s instruction. LLC cells were treated with different nanoparticles for 24 h. All cells were collected and washed twice with cold PBS, followed by incubation with 7-AAD-cy5.5 and Annexin V-FITC for 15 min at room temperature. The apoptosis was assessed by flow cytometry (BD FACSCanto II).

### Western blotting

Lung cancer cells were harvested and lysed using radioimmunoprecipitation assay buffer (Thermo Fisher Scientific, 89901, MA, USA) and Phenylmethylsulfonyl Fluoride (Beyotime, ST505, Shanghai, China) mixture (100:1 ratio) on ice for 30 min. Samples were centrifuged at 15000 rpm for 20 min; supernatant was added with loading buffer and heated at 100°C for 10 min. The samples were separated by SDS-PAGE and transferred to Polyvinylidene Difluoride membranes (Millipore, IPVH00010, MA, USA). The membranes were visualized by enhanced chemiluminescence (Millipore, WBKLS0100, MA, USA). The primary antibodies were as follows: Parp1(Proteintech, #13371), Bax (CST, #14796), Caspase 3 (CST, #14220), Tublin (CST, #2148).

### Fluorescence visualization

DiR-labeled NPs were used for intravital imaging and assessment of bio-distribution. Six-week-old Balb/c-Nude mice were immediately determined by fluorescent imaging after tail vein injection of DiR-labeled NPs. Time-lapse images were acquired continually within the initial 36 h after NPs injection.

### *In vivo* anti-tumor effects

Six-week-old Balb/c mice were subcutaneously implanted with 5 × 10^6^ LLC cells to establish a subcutaneous tumor model in mice. Ten days after inoculation, NPs were injected every other day for five times, and the NIR group was irradiated for 5 min after injection. The changes of body weight and tumor volumes were recorded. These mice were sacrificed on the 25 th day. The lung, heart, liver, spleen, kidney and tumor of the mice were taken for physiological sections.

### *In vivo* photothermal effect

Lung cancer mice model was established as mentioned above. NPs were injected by the tail vein. After 2 h, the mice were irradiated by 808 nm near-infrared light. The temperature changes of the tumors were observed by a photothermal imaging instrument.

### HE staining

Paraffin sections were stained with hematoxylin and eosin (HE) staining for morphometric analysis. Paraffin sections were dewaxed with different concentrations of xylene and alcohol. The slices were stained with hematoxylin staining solution (Harris) for 3 min and then differentiated with 0.8% hydrochloric acid. These slices were stained with eosin stain for 20 s, rinsed with water for 5 min and transparently sealed.

### Immunohistochemistry analysis

Immunohistochemical staining was performed on tissue slides using the streptavidin–biotin–peroxidase complex method. The procedure of antigen extraction was to place the sample in the antigen extraction solution containing 10 mM sodium citrate buffer and heat it in a pressure pot. Ki-67 antibody (CST, 1:200) and CD31 antibody (CST, 1:100) were used to detect. Slides were counterstained with hematoxylin.

### Statistical analysis

Statistical analysis was performed with GraphPad Prism 7.0 (CA, USA). Statistical significance was determined by one-way ANOVA or unpaired Student’s T-test. All data used in this study satisfied the assumptions of the statistical tests. All quantitative data were shown as the mean ± S.D. *P* values were indicated. *P *<* *0.05 was considered statistically significant.

## Results and discussion

### Preparation and characterizations of self-assembled nanoparticles

**Figure 1.**
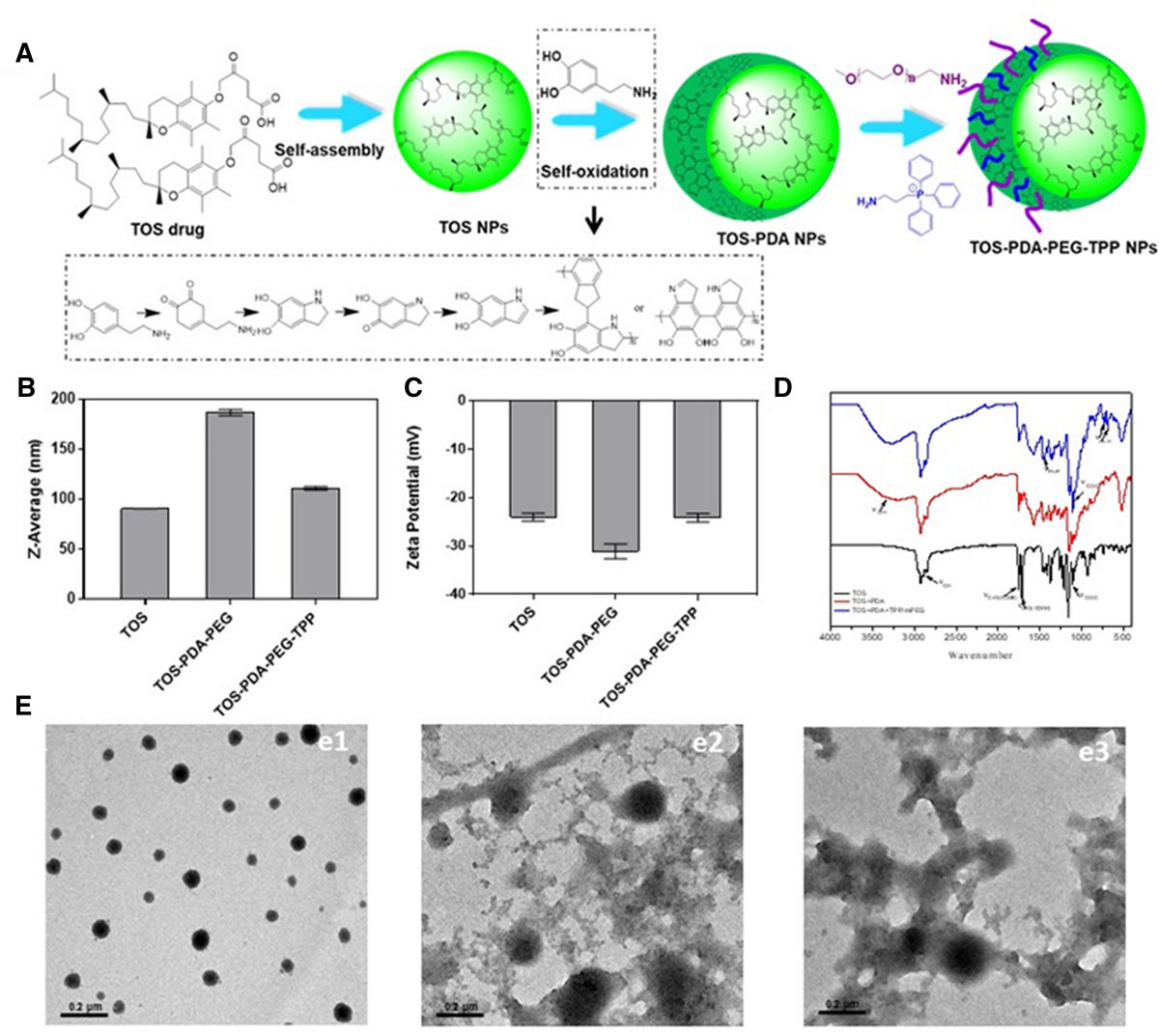
(**A**) The synthesis routes of mitochondria-targeting TOS-PDA-PEG-TPP NPs. (**B**) The size, (**C**) Zeta potential and (**D**) FTIR spectra of nanoparticles. (**E**) The morphologies of (e1) TOS NPs, (e2) TOS-PDA-PEG NPs, and (e3) TOS-PDA-PEG-TPP NPs.

To synthesize the nanoparticles, α-TOS was dissolved in DMSO solution to prepare nanoparticles (TOS NPs) by self-assemble. Then, the TOS NPs solution was mixed with Tris-HCl solution, and DA·HCl was added to form PDA-coated TOS-PDA NPs by self-oxidation polymerization. Mitochondrially targeted nanoparticles (TOS-PDA-PEG-TPP NPs) were finally obtained by adding TPP and mPEG-NH_2_ into the TOS-PDA NPs solution. The mitochondrial targeting TOS NPs synthesis pathway is shown in Fig. 1A. We observed that the particle sizes of nanoparticles were < 200 nm by particle size potentiometer (Fig. 1B). Compared with TOS NPs, the widths of TOS-PDA-PEG and TOS-PDA-PEG-TPP nanoparticles were slightly increased, which was an essential characterization of the successful modification of TOS NPs. It is well known that the particle size of the nano drug carrier affects its cumulative efficiency and endocytosis efficiency at the tumor sites. The nanocarrier with a particle size < 200 nm is more likely to reach the tumor site through the EPR effect. The zeta potentials of TOS, TOS-PDA-PEG and TOS-PDA-PEG-TPP nanoparticles were all distributed between –20 and –35 mV (Fig. 1C). The absorption peaks of functional groups were also apparent in their spectra (Fig. 1D). The absorption peak of TOS can be observed by UV-visible absorption spectroscopy (Supplementary Fig. S1). The peak of TOS-PDA-PEG and TOS-PDA-PEG-TPP have no apparent deviation, and the introduction of PDA and TPP has slight effect on the energy absorption of nanoparticles. TEM was used to observe the morphology of TOS, TOS-PDA-PEG and TOS-PDA-PEG-TPP. As shown in Fig. 1E, TOS formed spherical nanoparticles. When coated with PDA, TOS-PDA-PEG and TOS-PDA-PEG-TPP showed apparent membrane structure and uniform particle size distribution. Compared with single TOS, the particle size increased slightly, indicating that PDA was successfully coated on the surface of TOS. The results were consistent with the previous characterization data.

**Figure 2.**
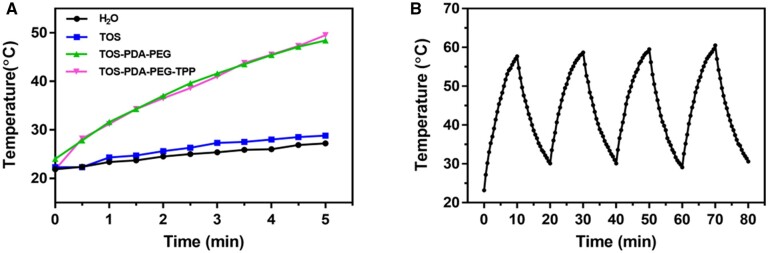
(**A**) Temperature changes of four groups of different materials after NIR irradiation. (**B**) Light stability of TOS-PDA-PEG-TPP for four consecutive exposures in 80 min.

As shown in Fig. 2A and B, compared with the control group H_2_O and TOS, the temperatures of TOS-PDA-PEG and TOS-PDA-PEG-TPP continue to rise, under the continuous irradiation with a near-infrared light for 5 min. The temperature of TOS-PDA-PEG-TPP has reached 50°C, which provides the necessary conditions for subsequent anti-tumor treatment. The strength of nanoparticles is a critical evaluation index of drug delivery *in vivo*, which plays a decisive role in the later anti-tumor effect. In this study, the nanoparticles were co-cultured with 5% serum to simulate the *in vivo* environment, and the changes in particle size and potential with time were observed (Supplementary Fig. S2). At the initial stage of co-culture, the particle size decreased slightly. With the increase of culture time, the particle size and the potential increased slightly. However, the particle size was stable, and the particle size was < 200 nm, indicating that the nanoparticles had good stability in the serum solution. The above results showed that the constructed TOS-PDA-PEG-TPP in this study had good stability and had the potential to prolong the circulation time in the blood, thereby increasing the amount of TOS-PDA-PEG-TPP reaching the tumor site.

### *In vitro* cellular uptake

**Figure 3.**
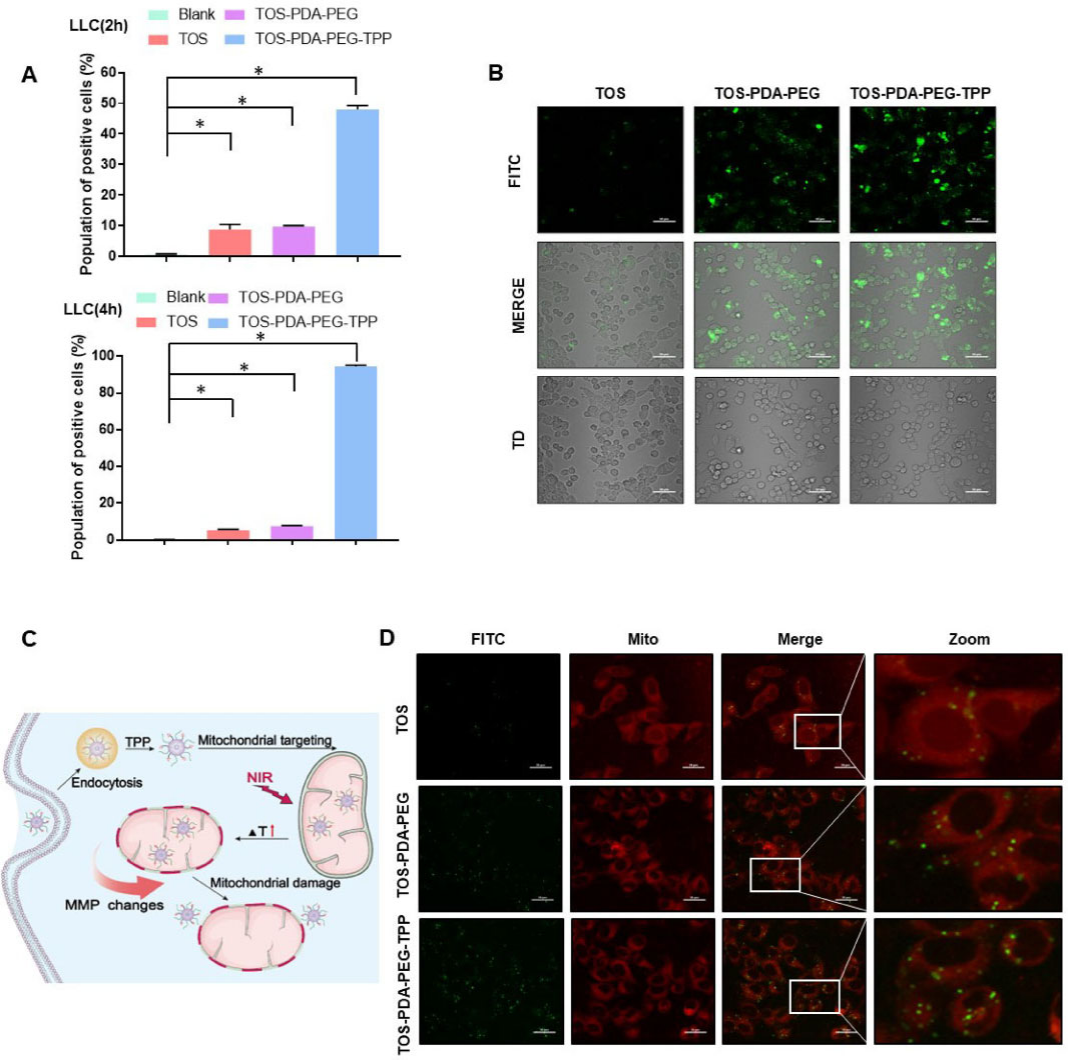
(**A**) Flow cytometry analysis of cellular uptake of indicated nanoparticles. (**B**) Confocal laser images of the intake of nanoparticles by LLC cells (4 h). (**C**) Schematic diagram of mitochondrial membrane potential damage caused by nanoparticles entering mitochondria. (**D**) Quantitative analysis (CLSM) of indicated nanoparticles entering mitochondria after 4 h co-culture with LLC cells.

To address whether the nanoparticles could be engulfed by tumor cells, we did flow cytometry and confocal laser scanning microscopy. Flow cytometry showed that both LLC and A549 cells could absorb FITC-labeled nanoparticles after co-culture for 2 h or 4 h, respectively (Fig. 3A, Supplementary Fig. S3A). Tumor cells showed more vital ability for TOS-PDA-PEG-TPP NPs uptake. Similar results were observed in LLC cells by confocal microscopy (Fig. 3B). Since TPP has been reported to have mitochondrial-targeting properties, we loaded TPP into the engineered nanoparticles to guide the drugs to mitochondria. To demonstrate whether TPP decorated NPs could realize mitochondrial targeting, we did confocal microscopy by staining the cells with mito-tracker and FITC-labeled nanoparticles treatment. Results showed that TOS-PDA-PEG-TPP nanoparticles increased mitochondrial accumulation compared to TOS NPs and TOS-PDA-PEG NPs (Fig. 3C and D). These results indicate that the designed nanoparticles can be well engulfed by tumor cells and realize mitochondrial-targeting ability effectively.

### *In vitro* anti-tumor effects of nanoparticles

**Figure 4.**
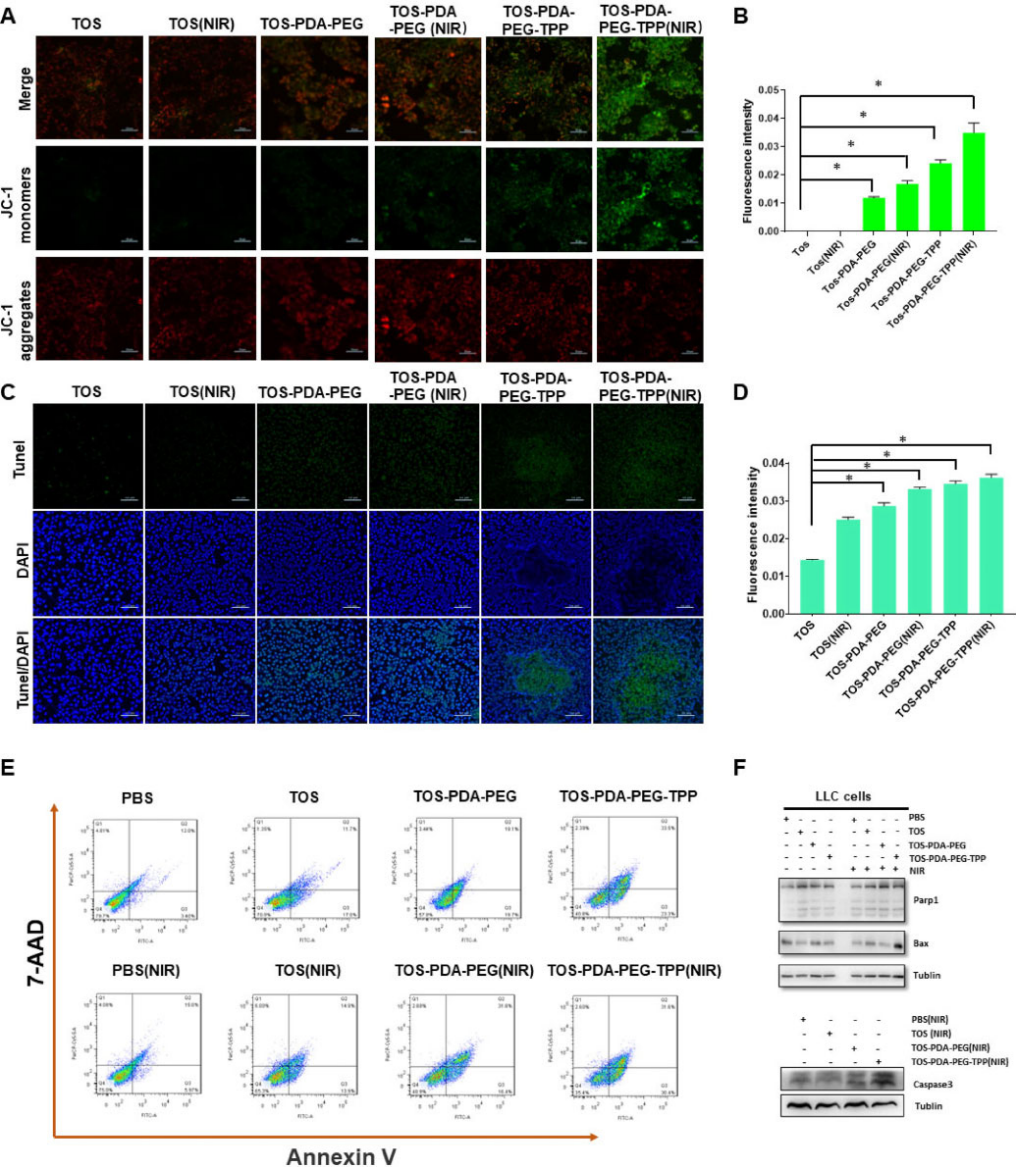
(**A**) Laser confocal images of indicated groups after JC-1 staining of 6 h co-culture (scale bar =50μm). (**B**) Semi-quantitative analysis of fluorescence intensity of JC-1 staining. (**C**) The confocal image of tunel after 24 h co-culture with indicated nanoparticles (scale bar = 50μm). (**D**) Semi-quantitative analysis of fluorescence intensity of tunel. (**E**) Flow cytometric analysis of annexin V and 7-AAD double staining after 24 h co-culture of indicated groups. (**F**) Protein levels of Parp1, Bax and Caspase 3 of indicated groups.

We conducted several assays to explore the anti-tumor effect of these nanoparticles. First of all, we detect the changes in mitochondrial membrane potential. Results showed that the membrane potential decreased significantly upon TOS-PDA-PEG-TPP NPs treatment and NIR irradiation (Fig. 4A and B). This may be result by the enhanced mitochondrial targeting ability of TOS-PDA-PEG-TPP NPs. It is well known that the decrease in mitochondrial membrane potential is a hallmark event in the early stages of apoptosis. We used confocal microscopy and flow cytometry to detect the early apoptosis of cancer cells treated with different nanoparticles. Confocal microscopy showed that TOS-PDA-PEG-TPP NPs combined with NIR treatment increased the apoptosis of cancer cells significantly (Fig. 4C and D). Western blot showed that the expressions of Parp1, Bax and Caspase 3 were increased dramatically in the TOS-PDA-PEG-TPP(NIR) group. However, NIR treatment alone did not induce apoptosis (Fig. 4E and F). We also carried out a cloning formation assay, and results showed that the TOS-PDA-PEG-TPP group could exert a better anti-cancer effect than TOS alone (Supplementary Fig. S4A). These results showed that the TOS-PDA-PEG-TPP (NIR) composite treatment could enhance the mitochondrial targeting impact of nanoparticles and promote cell apoptosis by changing the mitochondrial membrane potential.

### *In vivo* anti-tumor effects of different nanoparticles

**Figure 5.**
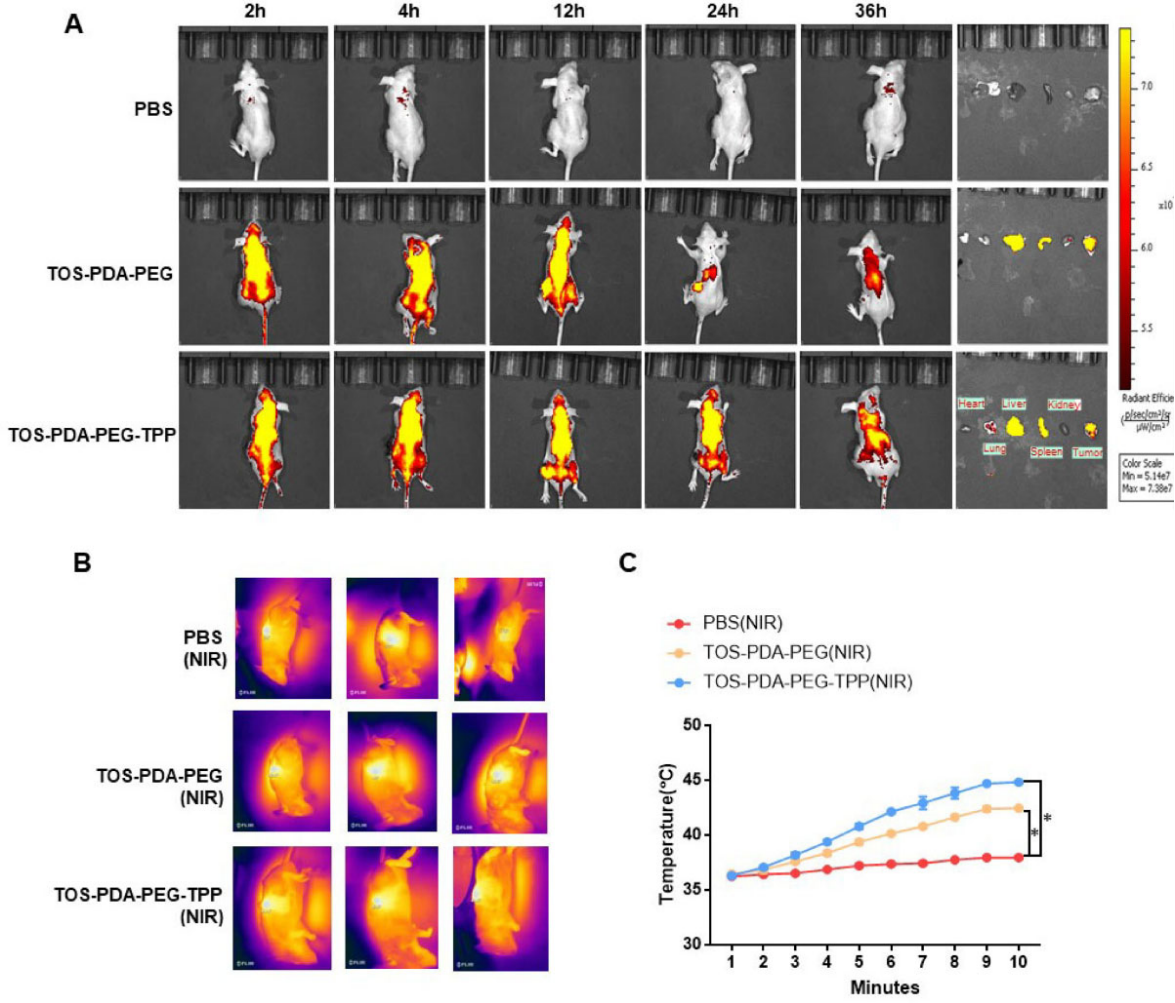
(**A**) Distribution of drugs in mice at different time points (2 h, 4 h, 12 h, 24 h, 36 h) after tail vein injection. (**B**) The temperature changes of mice after different drugs were injected into the tail vein and irradiated by NIR. (**C**) Temperature changes at the tumor site over 10 min.

To explore the tumor-targeting effect of nanoparticles, we injected them through the tail vein in a subcutaneous xenograft nude mouse model. Then, the *in vivo* distribution of nanoparticles was detected in the next 36 h using *in vivo* imaging technology. Results indicated that TOS-PDA-PEG-TPP was distributed throughout the body within the first 2 h after intravenous injection and then accumulated in the tumor sites effectively at 36 h (Fig. 5A). This result indicated that the nanoparticles loaded with TPP had accurate tumor targeting and could effectively transport the drug to the tumor sites of mice. In photothermal imaging, TOS-PDA-PEG-TPP reached 45°C in 10 min after near-infrared light irradiation. Results showed that the maximum temperature of drugs without TPP reached 42°C, resulting in corresponding lethal effects on tumors (Fig. 5B and C). It indicated that *in vitro* near-infrared irradiation combined with *in vivo* drug treatment ruptured the PDA coating membrane of nanoparticles and released drugs against tumor cells.

**Figure 6.**
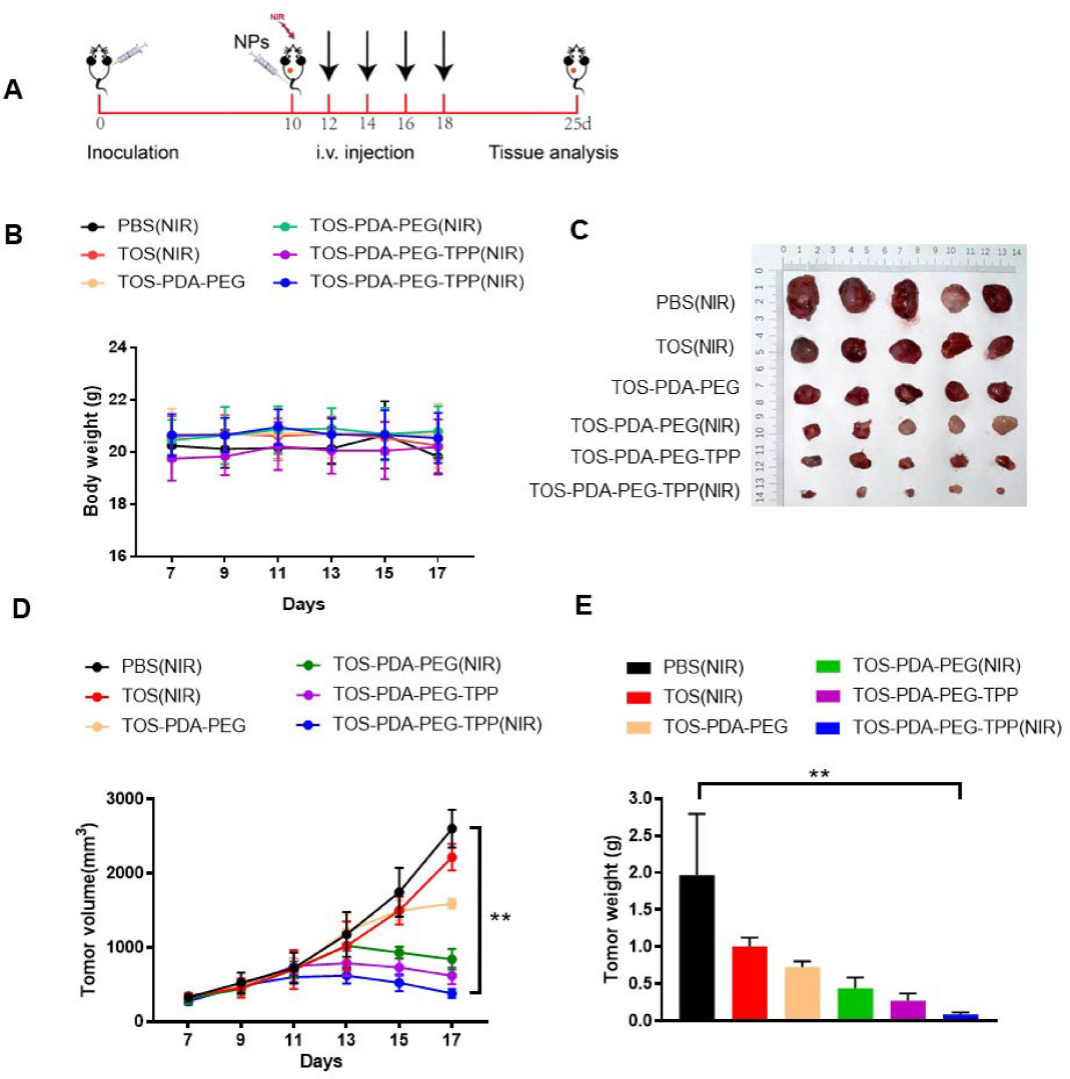
(**A**) Schematic diagram of tumor inoculation and administration in mice. (**B**) Body weight changes of mice in indicated groups after drug injection. (**C**) Tumor images of indicated groups of mice after treatment. (**D**) Changes in tumor volume in six groups during treatment. (**E**) Statistical map of tumor weight of six groups of mice after treatment.

To further explore the anti-tumor effect and side effects of nanoparticles *in vivo*, we established a subcutaneous tumor transplantation model in mice and explored the combined effect of nanocomposites and PTT. The mice were divided into six groups: PBS(NIR), TOS(NIR), TOS-PDA-PEG, TOS-PDA-PEG(NIR), TOS-PDA-PEG-TPP, TOS-PDA-PEG-TPP(NIR). As shown in Fig. 6A, we performed treatment experiments on the 10th day after inoculating subcutaneous xenografts. Nanoparticles were injected intravenously every 2 days, and NIR treatment was performed 2 h post-injection. The results showed no significant change in the body weight of the mice (Fig. 6B), TOS-PDA-PEG-TPP NPs combined with NIR treatment had the most excellent anti-tumor effects, and the subcutaneously transplanted tumor was significantly decreased (Fig. 6C–E).

**Figure 7.**
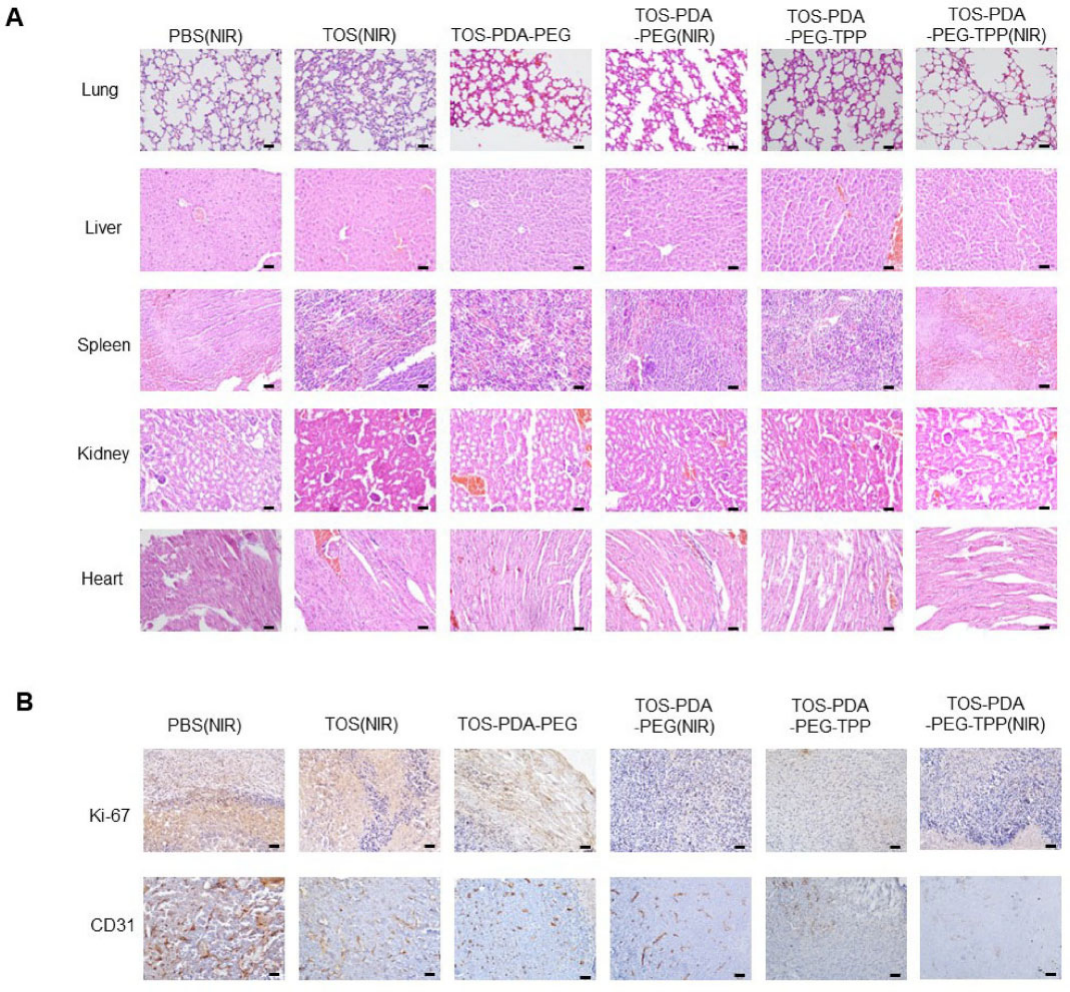
(**A**) HE staining (lung, liver, spleen, kidney, heart and tumor) of mice treated with indicated drugs (scale bar = 100 μm). (**B**) Immunohistochemical staining (Ki-67, CD31) of indicated mice treated with different drugs (scale bar =100 μm).

We further performed HE staining and the results showed no obvious damage to essential organs such as the heart, liver, spleen, etc., suggesting that the nanoparticles did not have noticeable side effects on animals (Fig. 7A). In addition, we performed immunohistochemical staining on tumor tissues to examine the expression levels of CD31 and Ki-67 in tumors. The results suggested that the expression levels of CD31 and Ki-67 in the TOS-PDA-PEG-TPP (NIR) group were significantly decreased, which indicated that nanoparticles could reduce substantially the angiogenesis and proliferation of subcutaneous xenograft tumors and had an excellent therapeutic effect (Fig. 7B). In conclusion, our experiments demonstrate that the designed nanoparticles have an anti-tumor effect in mice.

## Conclusion

In this study, we used a novel near-infrared laser-induced nanoparticle delivery system to prolong the blood circulation time of TOS NPs. Moreover, TPP was introduced as mitochondria-targeting drug to realize the subcellular localization to enhance the anti-tumor effect of TOS NPs. It not only solved the shortcomings of easy dissolution and poor photostability of TOS but also proved that the composite nanoparticles could accelerate the apoptosis of tumor cells by changing the mitochondrial membrane potential *in vitro* and *in vivo*. In addition, *in vivo* experiments showed that NIR-irradiated nanoparticles could rapidly rise tumor temperature, have excellent EPR effect and effectively reduce nanoparticle toxicity *in vivo*. In conclusion, the results have opened up a new route for the self-assembly of carrier-free nanoparticles based on PTT.

## Supplementary data

Supplementary data are available at *REGBIO* online.

## Supplementary Material

rbac051_Supplementary_Data
