# Supplementary material for: Mitochondria-targeting polydopamine-coated nanodrugs for effective photothermal- and chemo-synergistic therapies against lung cancer
Source: Regen Biomater. 2022 Aug 1;9:rbac051. doi: 10.1093/rb/rbac051 (PMC9362997; doi:10.1093/rb/rbac051)
Supplement: rbac051_Supplementary_Data [file rbac051_supplementary_data.docx]

**Supporting Information**

**Mitochondria-targeting Polydopamine-coated Nanodrugs for Effective Photothermal- and Chemo- Synergistic therapies Against Lung Cancer**

Ziyu Meng^1,2^, Binchao Wang^1^, Yiqiang Liu^2^, Yejian Wan^2^, Qianshi Liu^2^, Huasheng Xu^2^, Renchuan Liang^2^, Yin Shi^2^, Peng Tu^1*^, Hong Wu^2*^, Chuan Xu^2*^

^1^College of Science, Gansu Agricultural University, Lanzhou 730070, China;

^2^Integrative Cancer Center & Cancer Clinical Research Center, Sichuan Cancer Hospital&Institute, Sichuan Cancer Center, School of Medicine, University of Electronic Science and Technology of China, Chengdu, 610047 China.

Corresponding authors: [*tupeng815@163.com*](mailto:tupeng815@163.com)*(P.T);* [*wuhongzao@126.com (H.W)*](mailto:wuhongzao@126.com%20(H.W)); [*xuchuan100@163.com*](mailto:xuchuan100@163.com) *(C.X);*

**
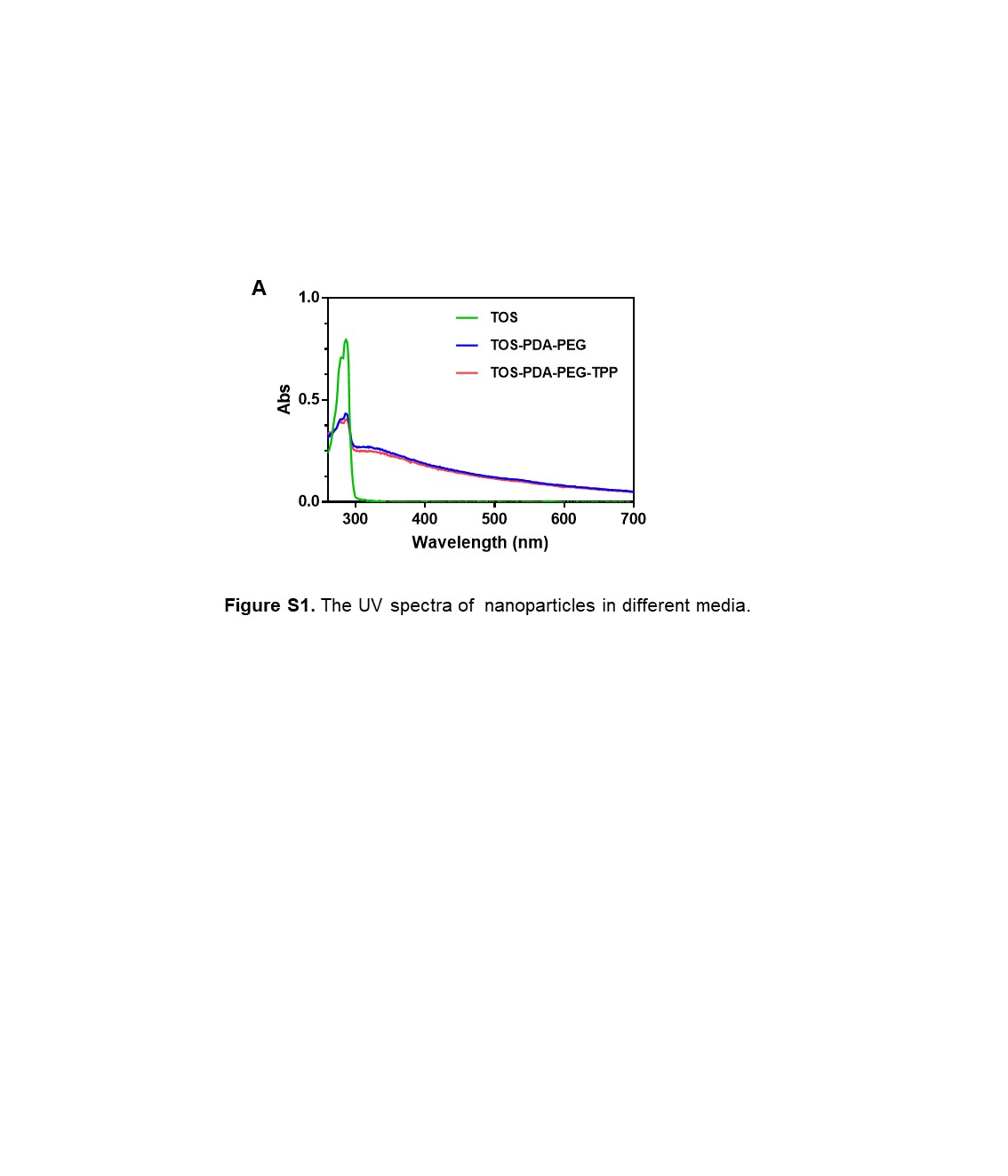
**

**Supplementary Figure 1.** (A) The UV spectra of TOS, TOS-PDA-PEG and TOS-PDA-PEG-TPP nanoparticles.

**
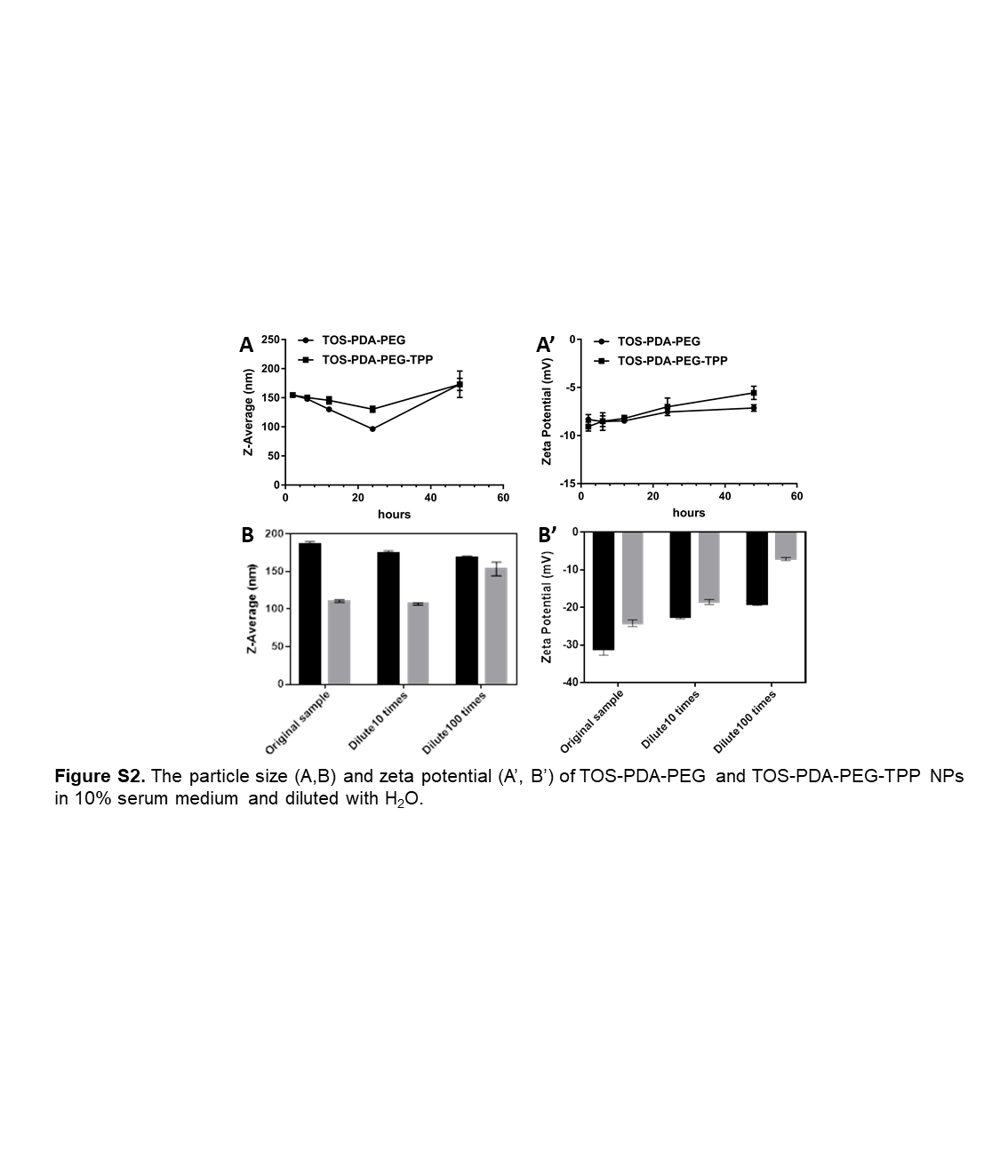
**

Supplementary Figure 2. The particle size (A, B) and zeta potential (A’, B’) of TOS-PDA-PEG and TOS-PDA-PEG-TPP NPs in 10% serum medium and diluted with H_2_O.

**
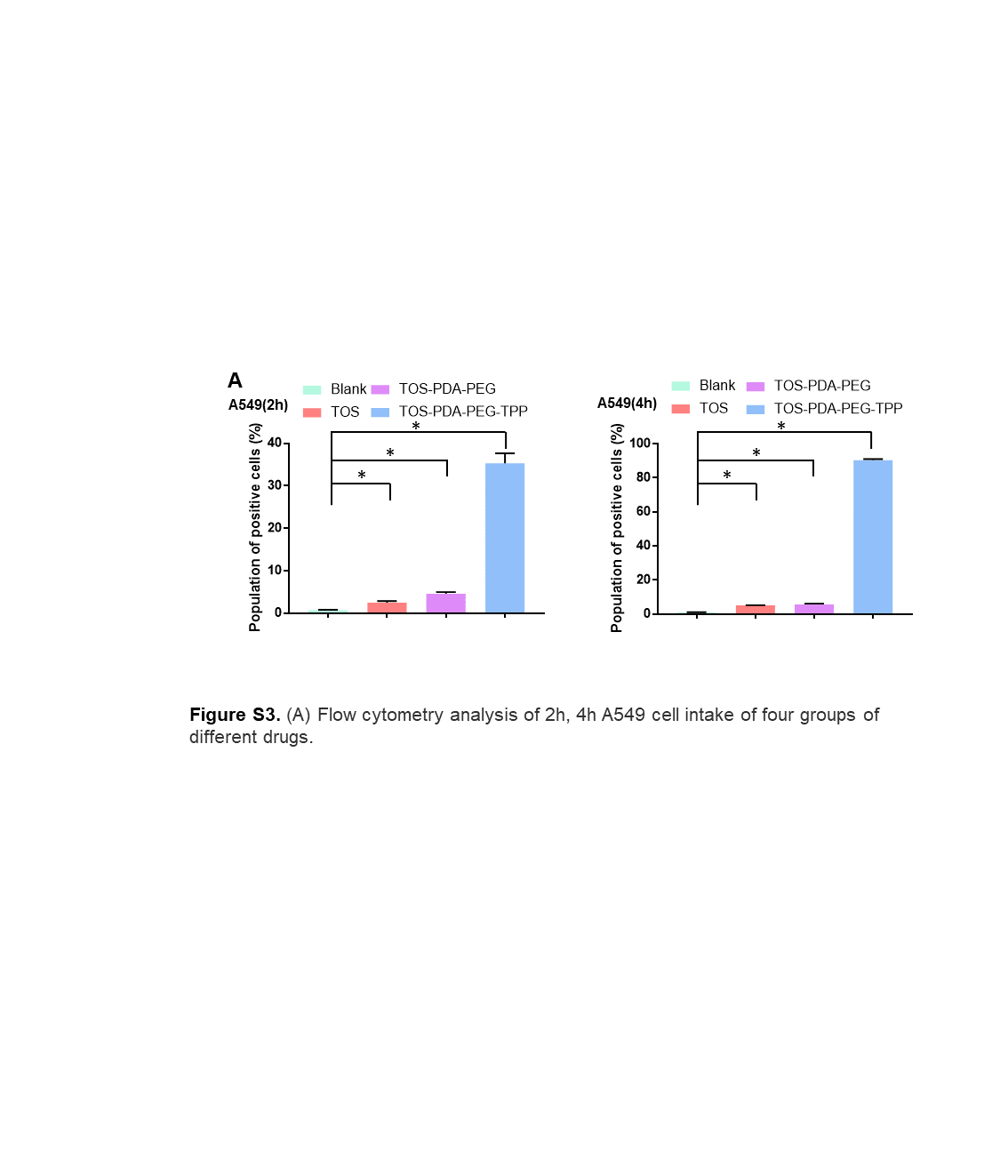
**

Supplementary Figure 3. (A) Flow cytometry analysis of 2 h, 4 h A549 cell intake of four groups of different drugs.


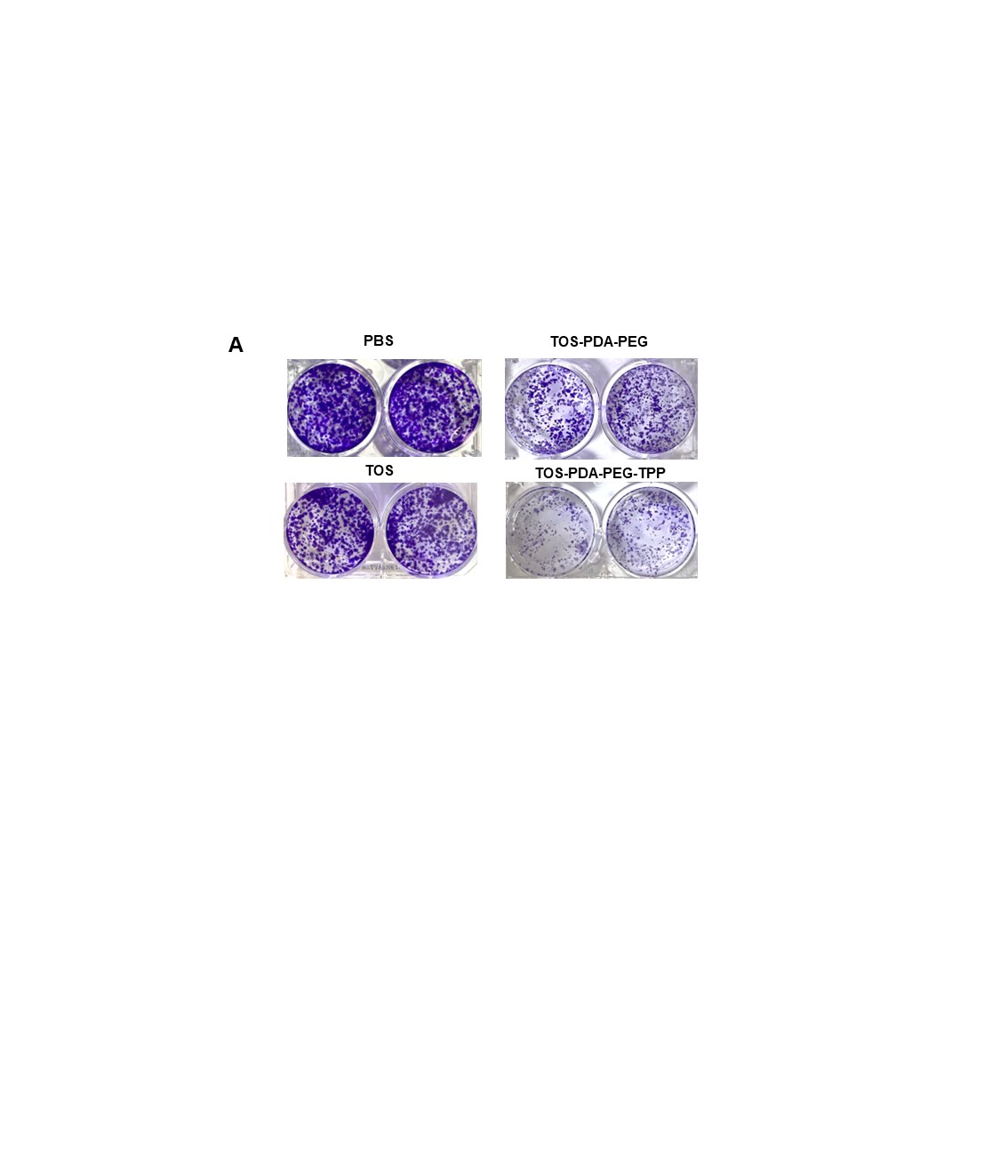


Supplementary Figure 4. (A) Colony formation of LLC cells treated with different samples.
